# Thermodynamic Modeling of Solvent-Assisted Lipid Bilayer Formation Process

**DOI:** 10.3390/mi13010134

**Published:** 2022-01-15

**Authors:** Hongmei Xu, Hyunhyuk Tae, Nam-Joon Cho, Changjin Huang, K. Jimmy Hsia

**Affiliations:** 1 School of Mechanical and Aerospace Engineering, Nanyang Technological University, Singapore 639798, Singapore; HONGMEI001@e.ntu.edu.sg; 2 School of Materials Science and Engineering, Nanyang Technological University, Singapore 639798, Singapore; HYUNHYUK001@e.ntu.edu.sg; 3 School of Chemical and Biomedical Engineering, Nanyang Technological University, Singapore 637459, Singapore

**Keywords:** supported lipid bilayer, thermodynamics, solvent exchange, self-assembly, SALB

## Abstract

The solvent-assisted lipid bilayer (SALB) formation method provides a simple and efficient, microfluidic-based strategy to fabricate supported lipid bilayers (SLBs) with rich compositional diversity on a wide range of solid supports. While various studies have been performed to characterize SLBs formed using the SALB method, relatively limited work has been carried out to understand the underlying mechanisms of SALB formation under various experimental conditions. Through thermodynamic modeling, we studied the experimental parameters that affect the SALB formation process, including substrate surface properties, initial lipid concentration, and temperature. It was found that all the parameters are critically important to successfully form high-quality SLBs. The model also helps to identify the range of parameter space within which conformal, homogeneous SLBs can be fabricated, and provides mechanistic guidance to optimize experimental conditions for lipid membrane-related applications.

## 1. Introduction

Supported lipid bilayers (SLBs) have received extensive attention from researchers due to their ability to faithfully mimic biological membranes [1,2,3,4]. SLBs can be used to investigate lipid membrane-mediated biological processes in vitro in a controlled manner [5,6,7], for biofunctionalization of inorganic solid substrates for biocompatibility [8,9], and in many other applications such as programmed drug delivery [10] and biosensing [11,12]. To realize their functionalities, versatile and intact SLBs must be reliably fabricated. Several techniques have been developed to fabricate SLBs, such as the Langmuir−Blodgett (LB) film technique [13], the spin-coating method [14], and the vesicle fusion (VF) method [12,15]. The LB technique relies on the transfer of lipid bilayers formed at the air-water interface to a solid substrate as it is vertically immersed into the liquid. This method is prone to producing low-quality SLBs with holes and decoupled leaflets, limiting their usefulness [16]. The spin-coating method can produce high-quality and defect-free lipid multilayers in dry conditions [17,18], but the multilayers’ stability deteriorates drastically upon hydration [19]. The VF method forms SLBs by allowing the lipid vesicles to adsorb onto the solid support, leading to vesicle rupture and membrane fusion [20]. Although the VF method has been widely employed, the use of preformed lipid vesicles makes the process complicated. In addition, the VF strategy requires fluid-phase lipids and thus the fabrication cannot be carried out below the phase transition temperature ($T_{m}$) of the lipids being used [21]. Moreover, spontaneous vesicle rupture is challenging for some substrate materials (e.g., gold [8] and titanium oxide [22]). Additional treatments are often needed to facilitate vesicle rupture on those substrates [23]. 

**Figure 1:**
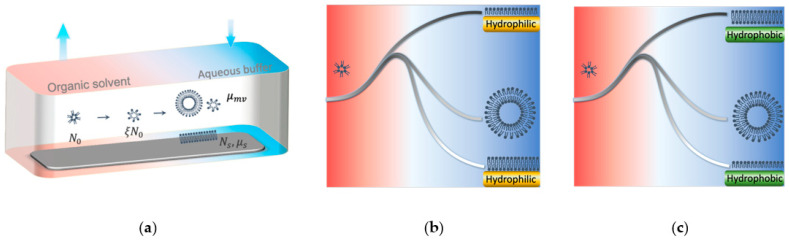
Schematic illustrations of Gibbs free energy profile along the solvent exchange coordinate path during the SALB formation process. (**a**) Schematic diagram of SLB formation pathway in the SALB method. (**b**) Expected Gibbs free energy profiles when inverted micelles disassemble and reorganize into different lipid structures in the system with a hydrophilic substrate during solvent exchange. (**c**) Expected Gibbs free energy profiles when inverted micelles disassemble and reorganize into different lipid structures in the system with a hydrophobic substrate during solvent exchange.

A recently developed strategy, termed the *solvent-assisted lipid bilayer* (SALB) formation [24,25,26,27], offers a simple, robust, and highly efficient protocol to fabricate SLBs. The substrate that needs to be coated is placed in a microfluidic chamber that contains phospholipids in a water-miscible organic solvent. As the organic solvent is progressively replaced by an aqueous buffer solution, SLBs form spontaneously on the solid substrate, as illustrated in Figure 1a. The design of the flow channel and the setup of the microfluidic system have been introduced in our previous publication [24]. This one-step SLB fabrication route [26] represents a more simplified workflow than existing methods, and works for a wider range of phospholipids and sterol compositions [28,29]. Since it does not depend on the quality of lipid bilayers at the water-air interface, or on the controlled rupture of lipid vesicles, this method effectively overcomes the technical difficulties of existing methods. It has been demonstrated that this method can be extended to coat SLBs on a variety of substrate materials. Previous experiments have identified several parameters that could affect the outcome of the SALB fabrication, including the lipid concentration in the organic solvent, substrate material, and temperature [24,27,30]. However, a fundamental understanding of the underlying physical mechanisms remains incomplete [31]. In this study, we estimate the energetics and carry out a thermodynamic analysis to investigate key parameters that affect the SALB formation process, including the substrate type, lipid concentration, and temperature. Such understanding provides guidance for optimizing the SALB protocol to ensure high-quality SLB fabrication.

## 2. SALB Formation–Estimation of Energetics and a Thermodynamics Model

### 2.1. Estimation of Energetics of SALB Formation

Our previous studies have demonstrated that formation of SLBs is highly dependent on the substrate material. Using the SALB method, SLBs can form on silicon dioxide and gold substrates [32], as shown in Figure 1b, but a lipid monolayer instead of SLB forms on alkanethiol-coated gold substrates as shown in Figure 1c. Silicon dioxide and gold substrates are hydrophilic while alkanethiol-coated gold substrate is hydrophobic, suggesting that lipid self-assembly depends on the hydrophilicity-hydrophobicity of the surface. 

Regulation of lipid organization through substrate hydrophobicity can be rationalized based on the Gibbs free energy profile of the SALB system [33]. As depicted schematically in Figure 1b,c, the Gibbs free energy changes as the lipids reorganize when the organic solvent is gradually replaced by an aqueous buffer (left to right in Figure 1). Prior to solvent exchange, lipids are presented as inverted micelles in the organic solution [34]. Injecting the aqueous buffer induces the rupture of inverted micelles as the exposure of hydrophobic hydrocarbon tails to water molecules would greatly increase the system energy, making the inverted micelles energetically unfavorable. The lipids may spontaneously self-assemble into various lipid structures, including lipid layers on the substrate, and conventional micelles and vesicles suspended in the aqueous buffer. On hydrophilic substrates (e.g., silicon dioxide and gold), the formation of lipid bilayers on the substrate surface prevents the exposure of hydrophobic tails to the hydrophilic environment – water molecules in the aqueous buffer and on the solid substrate surface. As a result, the system Gibbs free energy is significantly lower for lipid bilayer structures than for monolayers, i.e., $\mu_{sb}\ll \mu_{sm}$, where $\mu_{sb}$ and $\mu_{sm}$ are the chemical potentials of individual lipids in a supported bilayer and monolayer structures, respectively. Formation of suspended conventional micelles or vesicles also reduces the system Gibbs free energy by sealing the hydrophobic tails inside, i.e.,$\;\mu_{mv}<\mu_{sm}$, where $\mu_{mv}$ is the chemical potential of individual lipids in suspended conventional micelles or vesicles. Whether SLBs or suspended lipid structures are more energetically favored largely depends on the molecular structure of the lipid molecules and the substrate property [35]. For lipid molecules in the shape of cylinder (e.g., DOPC [36]) or truncated cone (e.g., DPPC and POPC [37]),$\;\mu_{sb}<\mu_{mv}$ and therefore they tend to form SLBs. In contrast, repulsive forces in the hydrophobic chains and headgroups will be generated from the steric, hydration, and electrostatic effects when lipid molecules in the shape of inverted cones form SLBs, giving rise to an imbalance of the lipid lateral pressure and elevated bending energy at the molecular level. However, on hydrophobic substrates (e.g., alkanethiol-coated gold, fluoropolymers [38], and the epoxy SU-8 [39]), $\mu_{sm}\ll \mu_{sb}$, inducing the formation of a supported lipid monolayer. This simple estimation of the Gibbs free energy indicates that SLBs form on hydrophilic surfaces and lipid monolayers form on hydrophobic surfaces.

### 2.2. Thermodynamic Analysis of SALB Formation

We consider the reaction chamber as a thermodynamic system, since the rate of aqueous buffer injection is much slower than the rate of lipid bilayer formation on the substrate. It is noted that the effect of solvent exchange kinetics is taken into consideration by introducing a scaling factor, ξ, which is defined as the ratio of the time average of the lipid concentration during solvent exchange (see Supplementary Materials) to the initial lipid concentration. In the reaction chamber in Figure 1a, the lipids initially in the form of inverted micelles in the solvent self-assemble into either supported lipid layers (a bilayer on hydrophilic substrates and a monolayer on hydrophobic substrates) or suspended lipid structures (i.e., conventional micelles and vesicles) after the system reaches equilibrium. Assuming that the total initial number of lipids in the reaction chamber is $N_{0}$, the number of lipids available for adsorption on the substrate is $\xi N_{0}$. Denoting the number of lipids in the supported lipid layer as $N_{s}$, the number of lipids re-assembled into suspended lipid structures is thus given by $N_{mv}=\xi N_{0}-N_{s}$. The system free energy is given as:

(1) $$G=N_{s}\mu_{s}+(\xi N_{0}-N_{s})\mu_{mv}-k_{B}T\left[\xi N_{0}\ln\left(\frac{\xi N_{0}}{N_{s}}\right)+(\xi N_{0}-N_{s})\ln\left(\frac{N_{s}}{\xi N_{0}-N_{s}}\right)\right]\;$$

where $\mu_{s}$ is the chemical potential of individual lipids in supported lipid layers, and $k_{B}$ and *T* are the Boltzmann constant and temperature, respectively. Minimizing the system free energy with respect to $N_{s}$ yields:

(2) $$N_{s}=\frac{\xi \cdot N_{0}}{1+exp\left(-\frac{\mu_{mv}-\mu_{s}}{k_{B}T}\right)}$$

## 3. Results

### 3.1. Lipid Concentration

In our earlier experiments [24,27,30,32], the amount of adsorbed phospholipids on the substrate was monitored in real time by using the quartz crystal microbalance-dissipation (QCM-D) monitoring technique [40,41], which allowed us to record the resonance frequency shift and measure lipid layer formation with nanoscale resolution. The relation between the lipid mass change and the measured frequency change is provided by the Sauerbrey equation: Δm=−C·Δf, where *C* is a constant related to the properties of the QCM-D quartz crystal sensor chip, Δm is the mass of adsorbed lipids per unit surface area, and Δf is the measured frequency shift [42]. The number of lipids in Equation (2) is first converted to the mass of lipids. This allows us to directly correlate the number of adsorbed lipids $N_{s}$ with the resonance frequency shift Δf. The SLB coverage percentage can be defined as the ratio of frequency change due to the current level of SLB coverage Δf to the frequency change due to a full-spanning SLB $\Delta f_{c}$:

(3) $$\Delta f/\Delta f_{c}=\left\{-\frac{V\cdot \xi }{C\cdot A\cdot \Delta f_{c}}\times \frac{1}{1+exp\left(-\frac{\mu_{mv}-\mu_{s}}{k_{B}T}\right)\;}\;\right\}\times c_{0}$$

where *V*, *A* and $c_{0}$ are the volume of the fluidic chamber, the active quartz crystal area, and the initial lipid mass concentration in the organic solvent, respectively. 

**Figure 2:**
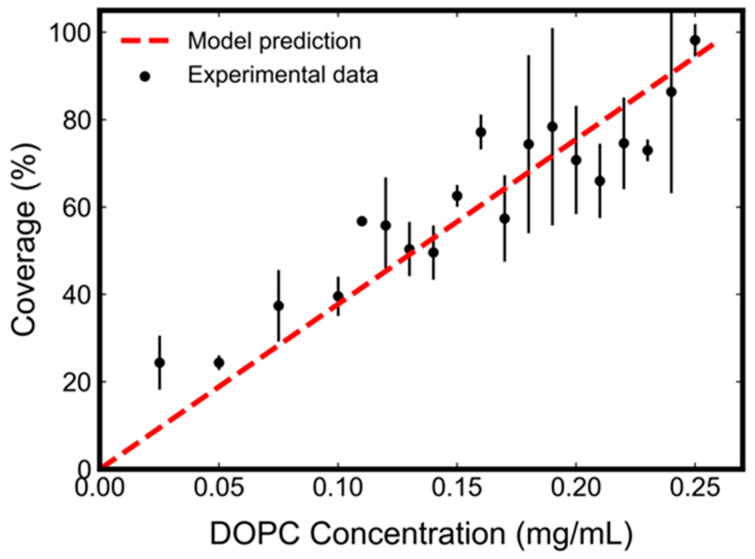
Effect of initial DOPC lipid concentration on SLB formation by the SALB method.

Our model predicts that the SLB surface coverage percentage is linearly dependent on the initial lipid concentration according to Equation (3). This prediction has been confirmed by our experimental characterization (black solid circles with error bars in Figure 2). Experiments were carried out at a fixed temperature of 24.0 ± 0.5 °C, with the initial DOPC lipid concentration ranging from 0.025 mg/mL to 0.25 mg/mL in isopropanol solution. A microfluidic chamber with a silicon-dioxide-coated sensor substrate was used. An aqueous buffer (10 mM Tris, 150 mM NaCl, pH 7.5) was slowly infused into the isopropanol solution containing lipids. Upon this solvent-exchange step, the formation of DOPC SLB (Δf) was measured by the QCM-D technique. The frequency shift of the complete DOPC SLB ($\Delta f_{c}$) formed in our experiments was measured to be -25 Hz. Figure 2 shows excellent agreement between the model prediction and the experimental data. Based on our experimental setup [31] and model assumption (see Supplementary Materials), we set $V=58\;{\mathrm{mm}}^{3}$, $C=17.7\;\mathrm{ng}\cdot {\mathrm{cm}}^{-2}\cdot {\mathrm{Hz}}^{-1}$, $A=97\;{\mathrm{mm}}^{2}$, and ξ=0.05. Linear least squares fitting the experimental data to Equation (3) predicts that $\mu_{mv}-\mu_{s}=0.585\;\mathrm{kJ}/\mathrm{mol}$. This is consistent with our understanding of energetics in Section 2.1 ($\mu_{sb}<\mu_{mv}$ for cylindrical lipids) and close to reported values [43].

### 3.2. Effect of Temperature

The SALB method overcomes the temperature limit of the traditional VF strategy and has been demonstrated to produce homogenous SLBs in both the gel and liquid phases. The effect of temperature on SLB production during the SALB formation process is not well characterized or fully understood. According to Equation (3), our model predicts that temperature may modulate SLB formation through three different aspects. First, it directly affects the thermal energy ($k_{B}T$) of the system. Second, it may affect the chemical potentials of the lipids ($\mu_{mv}$ and $\mu_{s}$). But the thermodynamics of general lipid self-assembly shows that micelles and SLBs are in thermodynamically equivalent states [44,45], suggesting that they have the same dependence on temperature and any resulting difference in $\mu_{mv}-\mu_{s}$ should be largely independent of temperature. In addition, temperature can modulate the frequency shift $\Delta f_{c}$ by altering lipid packing density:

(4) $$\Delta f_{c}=-\frac{2M}{N_{A}\cdot C\cdot a_{0}}$$

where M, $N_{A}$ and $a_{0}$ are the molecular weight of the lipids, the Avogadro’s constant, and the area per lipid (APL), respectively. 

In general, APL increases with temperature, since a higher temperature causes increased disorder of the lipid hydrocarbon chain [46]. It was shown that APL changes with temperature linearly as [47,48]:

(5) $$a_{0}=a_{m}+k_{A}\cdot \left(T-T_{m}\right)=\{\begin{array}{l} a_{m}^{g}-k_{A}^{g}\cdot \left(T_{m}-T\right),\;\;T<T_{m}\\ a_{m}^{f}+k_{A}^{f}\cdot \left(T-T_{m}\right),\;\;T\geq T_{m} \end{array}$$

where $T_{m}$, $a_{m}$ and $k_{A}$ are the phase transition temperature of the membrane, the APL at the phase transition temperature, and a phase-related coefficient, respectively. The superscript g denotes the gel phase and f denotes the fluid phase. Substituting Equation (5) into Equation (4), $\Delta f_{c}$ can be expressed as a function of temperature:

(6) $$\Delta f_{c}=\left(-\frac{2M}{C\cdot N_{A}}\right)\times \left(\frac{1}{a_{m}+k_{A}\cdot \left(T-T_{m}\right)}\right)$$

When a complete SLB forms, $\Delta f/\Delta f_{c}=1$ in Equation (3), from which we can obtain the minimum lipid concentration to achieve full SLB coverage as:

(7) $$c_{min}=\left(\frac{2M\cdot A}{N_{A}\cdot V\cdot \xi }\right)\times \left(\frac{1+exp\left(-\frac{\mu_{mv}-\mu_{s}}{k_{B}T}\right)}{a_{m}+k_{A}\cdot \left(T-T_{m}\right)\;}\right)$$

**Figure 3:**
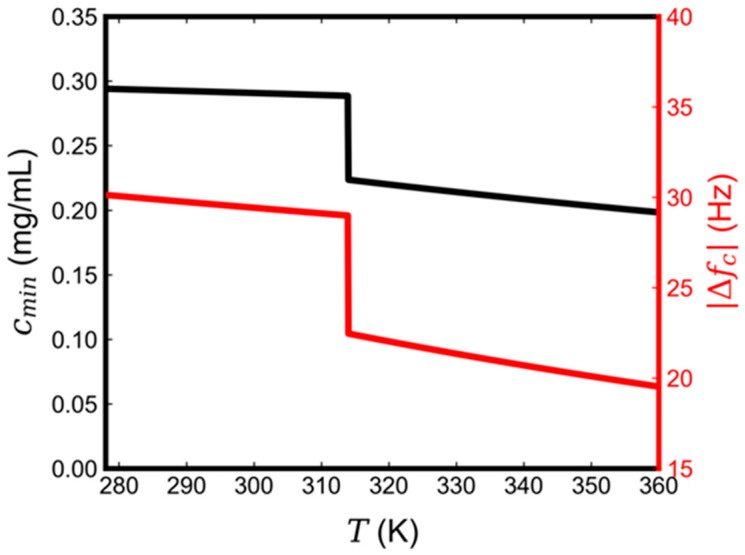
Effect of temperature during solvent exchange on the final frequency shift (**red**) and the minimum lipid concentration to form a complete DPPC SLB (**black**). The sudden drop is located at the phase transition temperature of DPPC, namely, $T_{m}=314\;K$.

Equations (6) and (7) enable us to predict the temperature effect on both the QCM-D frequency signal corresponding to a complete SLB and the minimum lipid concentration to form a complete SLB. Based on existing computational and experimental data for DPPC lipids, $M=734.039\;g\cdot \mathrm{mo}l^{-1}$, $T_{m}=314\;K$, $a_{m}^{g}=47.5\;{Å}^{2}$, $a_{m}^{f}=61.3\;{Å}^{2}$, $k_{A}^{f}\approx 0.2\;{Å}^{2}/℃$, $k_{A}^{g}\approx 0.06\;{Å}^{2}/{}^{\circ}C$ [47,48,49], and $\mu_{mv}-\mu_{s}\approx 1\;\mathrm{kJ}\cdot \mathrm{mo}l^{-1}$ [43]. Figure 3 shows that both $\Delta f_{c}\;$and $c_{min}$ monotonically decrease with the increase in temperature and follow a similar trend when the temperature is above or below $T_{m}$. At $T=T_{m}$, there is a sudden drop in both quantities due to the gel-to-liquid phase transition. The predicted drop in $\Delta f_{c}$ has been demonstrated in previous experiments [30]. In our experiments using 0.5 mg/mL DPPC in isopropanol, the final frequency shift upon complete SLB formation changed from −36.5 Hz in the gel phase at 297 K to −29.7 Hz in the liquid phase at 323 K, which represents a 21% surface mass density reduction.

### 3.3. Phase Diagram Prediction

A general expression of the SLB coverage percentage as a function of temperature and initial lipid concentration can be obtained by substituting Equation (6) into Equation (3):

(8) $$\Delta f/\Delta f_{c}=\left(\frac{N_{A}\cdot V\cdot \xi }{b\cdot M\cdot A}\right)\times \left(\frac{a_{m}+k_{A}\cdot \left(T-T_{m}\right)}{1+exp\left(-\frac{\mu_{mv}-\mu_{s}}{k_{B}T}\right)\;}\right)\times c_{0}$$

**Figure 4:**
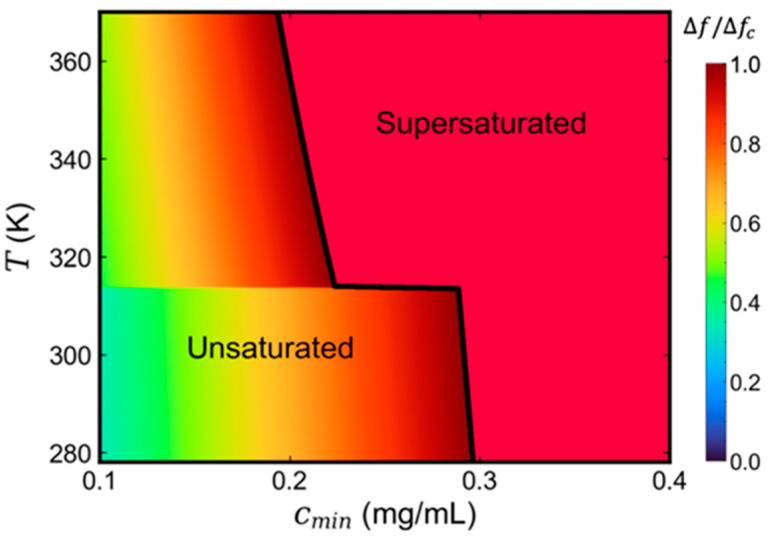
Phase diagram of the DPPC SLB coverage produced using the SALB method as a function of temperature and lipid concentration.

Equation (8) categorizes the parameters affecting the SLB coverage broadly into three groups: the one within the first bracket is a constant related to the chamber parameters, the flow rate, and the lipid type; the one in the second bracket is the temperature dependence, with two-stage behavior below or above the gel-to-liquid phase transition; and the third is the initial lipid concentration in the organic solvent. Equation (8) provides a quantitative way to evaluate the effects of various processing parameters on SLB formation using the SALB method. For instance, for the above described DPPC lipid system, the SLB coverage is primarily a function of two main parameters–temperature and initial lipid concentration. A phase diagram with a DPPC SLB coverage heatmap can thus be obtained, as shown in Figure 4. Phase diagrams for different organic solvent types and lipid compositions can readily be obtained if their effects on lipid chemical potentials and phase transition temperature can be quantitatively characterized. Figure 4 indicates that, at very low initial DPPC lipid concentrations, a complete SLB layer cannot form due to lack of lipid supply, while supersaturated SLBs are formed at very high initial lipid concentrations, leading to possible nonuniform lipid aggregates on the substrate [30]. The phase diagram provides a useful guideline to quantitatively identify the conditions within which high-quality, uniform, and intact SLBs can be achieved using the SALB method.

## 4. Conclusions

In this study, we have developed a thermodynamics model and carried out experimental verification of SLB formation via the SALB method. We particularly focused on the effects of the substrate surface properties, the initial lipid concentration in organic solvents, and the temperature on SLB formation to gain fundamental understanding of the underlying physicochemical mechanisms. The energetic estimate of the hydrophobic-hydrophilic properties of the substrate provides insights into the driving force of supported lipid mono-/bi-layer formation. The thermodynamic modeling leads to a general equation of the SLB coverage fraction as a function of various experimental parameters, including area per lipid, initial lipid concentration, and temperature. The model can be applied to both the gel phase and the liquid phase. One important conclusion of the current study is that there seems to exist a rather narrow range of the initial lipid concentration, below which the SLB would not fully cover the substrate surface and above which an over-confluent SLB layer with additional lipid aggregates could form, compromising the SLB quality. Important, highly useful, and quantitative information is summarized into a phase diagram. This diagram can guide the selection of various parameters to ensure quality SLB fabrication. This study has deepened the fundamental understanding of the SLB formation process, and broadened the applicability of this technique by expanding the parameter space for fabricating high quality SLBs.

## Data Availability

The data presented in this study are available in article.

## Supplementary Materials

The Supplementary Materials are available at https://www.mdpi.com/article/10.3390/mi13010134/s1.
