# Supplementary material for: Thermodynamic Modeling of Solvent-Assisted Lipid Bilayer Formation Process
Source: Micromachines (Basel). 2022 Jan 15;13(1):134. doi: 10.3390/mi13010134 (PMC8777629; doi:10.3390/mi13010134)
Supplement: Supplementary file 1 [file micromachines-13-00134-s001.zip › micromachines-1543939-supplementary.pdf]

micromachines

MDPI

Supplementary Materials

Thermodynamic Modeling of Solvent-Assisted Lipid Bilayer Formation Process

Hongmei Xu [1], Hyunhyuk Tae [2], Nam-Joon Cho [2,*], Changjin Huang [1,*] and K. Jimmy Hsia [1,3,*]

[1] School of Mechanical and Aerospace Engineering, Nanyang Technological University, Singapore 639798, Singapore; HONGMEI001@e.ntu.edu.sg
[2] School of Materials Science and Engineering, Nanyang Technological University, Singapore 639798, Singapore; HYUNHYUK001@e.ntu.edu.sg
[3] School of Chemical and Biomedical Engineering, Nanyang Technological University, Singapore 637459, Singapore
* Correspondence: njcho@ntu.edu.sg (N.-J.C.); cjhuang@ntu.edu.sg (C.H.); kjhsia@ntu.edu.sg (K.J.H.)

Citation: Xu, H.; Tae, H.; Cho, N.-J.; Huang, C.; Hsia, K.J. Thermodynamic Modeling of Solvent-Assisted Lipid Bilayer Formation Process. *Micromachines* **2022**, *13*, 134. https://doi.org/10.3390/mi13010134

Academic Editor: Toshihisa Osaki

Received: 22 December 2021
Accepted: 11 January 2022
Published: 15 January 2022

Publisher's Note: MDPI stays neutral with regard to jurisdictional claims in published maps and institutional affiliations.

Copyright: © 2022 by the authors. Submitted for possible open access publication under the terms and conditions of the Creative Commons Attribution (CC BY) license (https://creativecommons.org/licenses/by/4.0/).

Calculation of the scaling factor ξ

In our model, we consider the supporting lipid bilayer (SLB) formation via lipid self-assembly as a thermodynamic process. However, the solvent exchange process reduces the total number of lipids available to self-assemble. Assuming that the organic solvent and the injected aqueous buffer are instantly and completely mixed in the chamber, the bulk lipid concentration evolves as [1]:

$$c_b = c_0 \exp(-\frac{Q}{V}t) \quad \text{(S1)}$$

where c_0, Q, and V are the initial lipid concentration in the organic solvent before the aqueous buffer injection, the solvent exchange flow rate, and the volume of the chamber, respectively. The scaling factor ξ is deduced by taking the average of the lipid bulk concentration within the time range of lipid formation. It is known that, only when the water fraction ψ exceeds a critical value ψ_c, the lipids start to form conventional micelles and self-assemble into SLBs on the substrate, which corresponds to the time t_c. When the lipid concentration becomes so low that the reaction quotient equals to the chemical equilibrium constant, the lipid adsorption will stop. We assume the water volume fraction is ψ_k when SLB formation stops, and the corresponding timepoint is t_k. The corresponding water fractions at $t = t_c$ and $t = t_k$ are given by:

$$\begin{aligned} \psi_c &= 1 - \exp\left(-\frac{Q}{V}t_c\right) \\ \psi_k &= 1 - \exp\left(-\frac{Q}{V}t_k\right) \end{aligned} \quad \text{(S2)}$$

Hence, the time average of lipid bulk concentration can be calculated as:

$$\bar{c_b} = \frac{\int_{t_c}^{t_k} c_0 \cdot \exp(-\frac{Q}{V}x) \cdot dx}{t_k - t_c} = \frac{(1-\psi_c) - (1-\psi_k)}{\ln(1-\psi_c) - \ln(1-\psi_k)} \cdot c_0 \quad \text{(S3)}$$

The scaling factor ξ is defined as:

$$\xi = \frac{\bar{c_b}}{c_0} = \frac{(1-\psi_c) - (1-\psi_k)}{\ln(1-\psi_c) - \ln(1-\psi_k)} \quad \text{(S4)}$$

The measured ψ_c is in the range of 0.4 - 0.8 [2]. Taking $\psi_c = 0.7$ and $\psi_k = 0.001$, we obtain $\xi \approx 0.05$.

References

1. Bird, R.B.S.W.E.L.E.N. *Transport phenomena*; 2007.

2. Hohner, A.O.; David, M.P.C.; Rädlera, J.O. Controlled solvent-exchange deposition of phospholipid membranes onto solid surfaces. *Biointerphases* **2010**, *5*, 1-8, doi:10.1116/1.3319326.
